# Conditions for Thermoelectric Power Factor Improvements upon Band Alignment in Complex Bandstructure Materials

**DOI:** 10.1021/acsaem.4c02747

**Published:** 2025-01-24

**Authors:** Saff E. Awal Akhtar, Neophytos Neophytou

**Affiliations:** School of Engineering, University of Warwick, Coventry CV4 7AL, U.K.

**Keywords:** thermoelectric materials, theory and simulation, thermoelectric power factor, multiband model, band alignment, band convergence

## Abstract

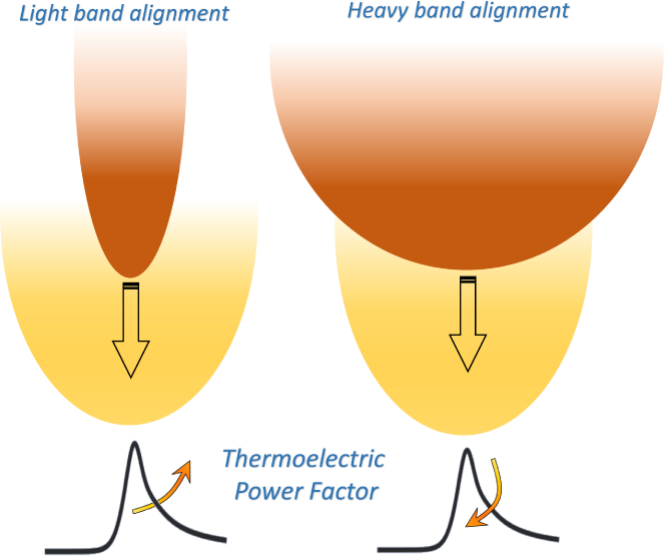

Band alignment (or band convergence) is a strategy suggested to provide improvements in the thermoelectric power factor (PF) of materials with complex bandstructures. The addition of more bands at the energy region that contributes to transport can provide more conducting paths and could improve the electrical conductivity and PF of a material. However, this can lead to increased intervalley scattering, which will tend to degrade the conductivity. Using the Boltzmann transport equation (BTE) and a multiband model, we theoretically investigate the conditions under which band alignment can improve the PF. We show that PF improvements are realized when intraband scattering between the aligned bands dominates over interband scattering, with larger improvements reached when a light band is brought into alignment. In the more realistic scenario of intra- and interband scattering coexistence, we show that in the light band alignment case, possibilities of PF improvement are present even down to the level where the intra- and interband scattering are of similar strength. For heavy band alignment, this tolerance is weaker, and weaker interband scattering is necessary to realize PF improvements. On the other hand, when interband scattering dominates, it is not possible to realize any PF improvements upon band alignment, irrespective of bringing a light or a heavy band into alignment. Overall, to realize PF improvements upon band alignment, the valleys that are brought into alignment need to be as electrically conducting as possible compared to the lower energy base valleys and interact as little as possible with those.

## 1 Introduction

Thermoelectric (TE) materials convert heat into electricity and vice versa and could be used for power generation as well as cooling applications.^1−6^ As such, they could contribute to energy sustainability and reduction of the use of fossil fuels. Their efficiency is quantified by the dimensionless figure of merit, *ZT* = σ*S*^*2*^*T*/*κ*, where *S* stands for the Seebeck coefficient, σ for the electrical conductivity, *T* for the absolute temperature, and κ for the thermal conductivity. The product σ*S*^2^ is called the power factor (PF) and quantifies the power production of the process. For the last two decades, the focus of the TE research targeted thermal conductivity reductions, and with the successful implementation of nanostructuring, large *ZT* improvements have been reached.^4−10^ The *ZT* has reached values of over 2 in many material cases and temperatures, doubling its values from around unity for the handful of materials of interest (Bi_2_Te_3_, PbTe, SiGe) as of a decade ago.^11−14^ These achievements expanded the material space exploration to many, more abundant, inexpensive, and environment friendly materials such as Half-Heusler alloys,^15−20^ selenides and tellurides,^21−24^ clathrates,^25,26^ skutterudites,^27−30^ etc.

Similar order of benefits from the PF, however, were not achieved, because the Seebeck coefficient (*S*) and electrical conductivity (σ) are adversely interconnected, and optimizing one frequently has a negative impact on the other. On the other hand, novel approaches related to band engineering have allowed for some PF improvements. With bandstructure engineering one alters the electronic structure of the material typically through alloying, to realize ideal circumstances for a high Seebeck coefficient and electrical conductivity, an effort to relax the adverse interdependence of the two quantities. Typically, modern TE materials have rich electronic structures, which consist of many bands and valleys, of varying degeneracies and effective masses, which allow for such design approaches.^31−35^ The most followed direction for PF improvement is band alignment (also referred to as band convergence). This refers to shifting the relative energy locations of the different valleys of the different bands to achieve energy alignment, thus increasing the number of channels available for transport.^36−40^ It can prove beneficial in two ways: the many aligned valleys transport channels can improve the carrier density and conductivity, but they also increase the density of states at the band edge which increases the Seebeck coefficient, both of which can lead to PF improvements.^37,41−44^

The complexity of the electronic structure can offer such PF possibilities; however, complex electronic structures also involve complex electronic scattering physics, some of which can be detrimental to the PF.^35,45−49^ An important scattering aspect is interband/valley scattering, under which carriers from one band/valley can scatter into another (into a band/valley that is brought into alignment, for example). In this case, although the number of transport bands/valleys increases, carrier scattering can also increase, and the benefits of alignment are reduced.^37,45,50^ Beyond scattering details, prior computational studies have also suggested that the attributes of the initial lower energy valley (base valley) with respect to the valley(s) that are brought into alignment (aligned valleys), also matter, with more benefits realized when light aligned valleys are introduced.^51,52^ On the other hand, the base and aligned valleys also have in general different degeneracies and different scattering details in between themselves as well. Thus, in order to have *a priori* indication if a given electronic structure will potentially lead to PF improvements upon band alignment through alloying or strain processes, the following questions need to be answered: (i) at what degree does the interplay between intra- versus intervalley scattering allows for PF improvements? (ii) what ratios between the effective masses of the base and aligned valleys are beneficial? (iii) how does the degree of valley degeneracy of the base or the aligned bands affect PF improvements?

In this paper, we employ a multiband parabolic model and the Boltzmann Transport Equation (BTE) including energy dependent scattering rates, with the goal of answering the questions posed above. We aim to provide a full investigation of the bandstructure and scattering parameter landscape that allows for PF improvements upon band alignment in complex bandstructure TE materials. We show that in general, PF improvements can only be realized when: (i) intravalley scattering dominates over intervalley scattering, and (ii) those benefits are larger when the aligned valley has a lighter effective mass. We also show that in the best case of realistic scenarios, such benefits can only lead to around doubling the PF, which is still important. In general, we suggest that for PF improvements, the aligned valleys need to be as conducting as possible compared to the base valleys, and interact as little as possible between themselves, and between the base valleys. Our results narrow down the design window for PF improvements, which would be highly beneficial for aiding experimentalists to target more effectively materials that can actually provide improvements. On a more optimistic note, it is suggested that some of the best new generation TE materials are polar optical phonon scattering limited or ionized impurity scattering limited.^49,53^ Since these are primarily anisotropic, intravalley scattering mechanisms, these stringent criteria are somewhat weakened.

The paper is organized as follows. In Section 2 we describe the methodology used to calculate the PF using a parabolic multiband model within the Boltzmann Transport Equation (BTE) formalism. In Section 3 we present our results and discuss the case of band alignment of single valley band under different scattering conditions. In Section 4 we present our results for band alignment of multivalley bands. Finally, in Section 5 we conclude.

## 2 Methodology

We have used a multiband parabolic effective mass model to study the effect of band alignment under different scattering considerations. The Boltzmann transport equation (BTE) theory under the energy dependent relaxation time approximation was used to evaluate the transport coefficients. The thermoelectric coefficients i.e., electrical conductivity (σ), Seebeck coefficient (*S*), and power factor (PF) at a particular Fermi level, *E*_F_, are computed as^54,55^
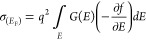
1
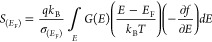
2
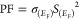
3

Above *q* is the electronic charge, *f* is the Fermi distribution function, *k*_B_ is Planck’s constant, *E*_F_ is the Fermi energy level, and *G*(*E*) is the transport distribution function (TDF), defined as
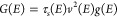
4where *v* is the bandstructure velocity, *g* is the density of states, and τ_*s*_ is the scattering (momentum) relaxation time. The band velocity and density of states are directly extracted from the *E(k)* relationship,  as
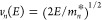
5
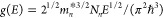
6where  is the mass, and *N*_*n*_ is the degeneracy of the *n*^th^ band. The scattering time in eq 4 is given by^54^
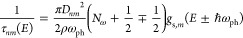
7where we consider a single electron-optical phonon process, which typically play dominant part in intervalley processes. These are central in our analysis below (by being able to support transitions of large energy and momentum exchange), but without loss in generality. Above, *g*_s,*m*_ is the scattering density of states (that carriers scatter into) for absorption and emission, *N*_*ω*_ is the Bose–Einstein distribution function, and ω_ph_ is the frequency of the phonons, for which we use ω_ph_ = 0.03 eV, typical for TE materials i.e. half-Heusler alloys.^50^*D*_*nm*_ is the inelastic optical deformation potential that defines the optical phonon scattering strength. In this work we use values from 1.2 × 10^10^ (eV/m) to 12 × 10^10^ (eV/m), again typical for semiconductors and TE materials.^50,53^ The subscripts *n* and *m* correspond to the different bands that interact through the scattering process. We consider both, intraband/valley, as well as interband/valley transitions, which can be facilitated by different deformation potentials. In the first case, *m* = *n* and it results to τ_*nn*_ with *D*_*nn*_ (which we will refer to as *D*_intra_), where in the second case *m ≠ n*, which results to τ_*nm*_ with *D*_*nm*_ (which we will refer to as *D*_inter_). The overall scattering rate from an initial state at energy *E* and band/valley *n* is then computed using Matthiessen’s rule as
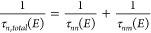
8

**Figure 1:**
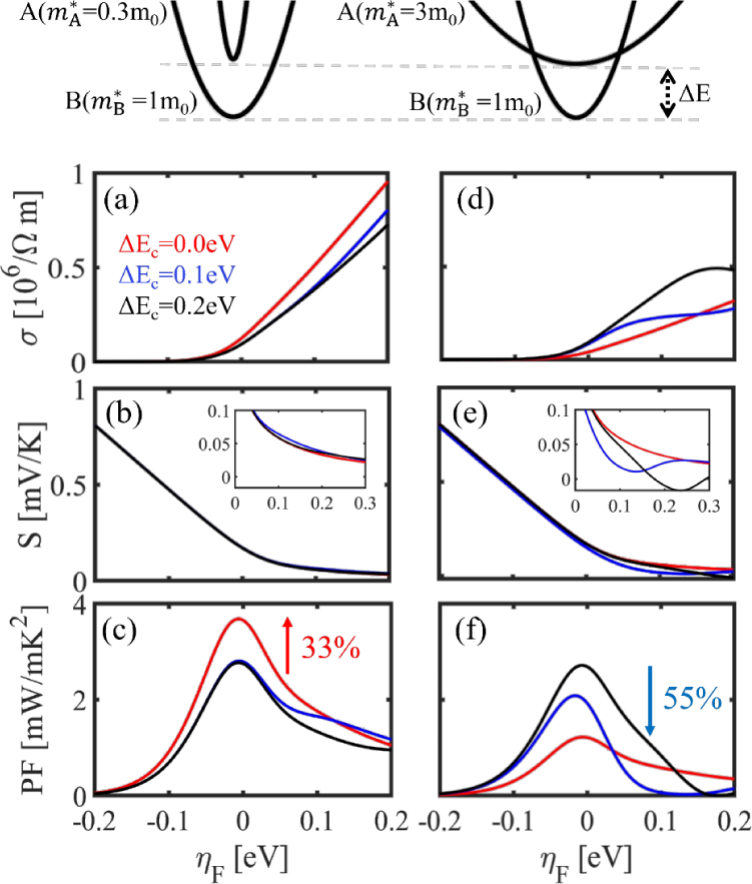
Band alignment illustrated for: aligning a light effective mass band and aligning a heavy effective mass band as shown in the top schematics. The corresponding transport coefficients, conductivity, Seebeck coefficient, and PF versus Fermi level, *E*_F_ are indicated in each column, (a–c) and (d–f) respectively. Here  is the effective mass of the base band (B) while  is the effective mass of the aligned band (A) in the unit of m_0_. The PF percentage change upon band alignment for the two cases is noted. The insets show a zoom-in of the Seebeck coefficient at high *E*_F_, where the PF peaks. We set *E* = 0 eV and η_F_ = 0 eV at the base band edge.

In Figure 1 we provide an illustrative example of the studies and analysis that follows. In the presence of intra/interband scattering, full band alignment of two valleys that are initially separated in energy affects the TE coefficients by increasing the number of conducting channels, but also the scattering states. We label the lower energy band as the “Base-B” band and the aligned band as the “Aligned-A” band. Figure 1a–c (left column) shows the effect on the TE coefficients (electrical conductivity, Seebeck coefficient and PF) upon *aligning a light band*. As the band aligns from Δ*E* = 0.2 eV down to Δ*E* = 0 eV, the electrical conductivity slightly increases due to the more conducting light band that now participates in transport, whereas the Seebeck coefficient remains unaffected since it is determined at first order by the band edge of the lowest band. Thus, a 33% PF improvement is observed. However, *aligning a heavy band*, as shown in Figure 1d–f (right column), is detrimental to the PF with a 55% reduction upon full band alignment, due to increase in scattering that the base band carriers experience into the large DOS of the heavy/low-conducting aligned band. Thus, band alignment is not always beneficial, and in fact in the latter case it should be avoided.^48,51^ Below we present a full analysis of when band alignment can, and when it cannot be beneficial.

Note that in both cases, the dominant factor that determines the change in the PF is the electronic conductivity, whereas the Seebeck coefficient has less of an effect. In fact, as shown in the insets in Figure 1, the Seebeck coefficient as expected follows the inverse trend compared to conductivity, and consequently the inverse trend compared to the PF as well. Also note that in our data for the Seebeck coefficient in Figure 1, the Seebeck coefficient only slightly shifts and that is only observed at high *E*_F_. Typically, one might expect that once an additional band is aligned, and the density of states (DOS) at the band edge increases, it will result in an increase in the Seebeck coefficient. Note, however, that this behavior is only observed when the Seebeck coefficients are plotted as a function of density. We present these figures in the Supporting Information (Figures S4.1 and S4.2) together with appropriate discussions. We show that, clearly, if plotted as a function of density, the entire Seebeck coefficient line, upon full band alignment acquires a right shift, indicating an increase in DOS (in a similar way to the typical interpretation of right shifts in the Pisarenko plot as increase in the effective mass). This is evident more in the case of heavy aligned band which increases the DOS substantially, compared to the case of the light aligned mass which does not cause significant changes in the DOS. On the other hand, PF improvements are reached in the latter case of light aligned band, clearly indicating that the conductivity is that determines the improvements. Also note that the conductivity will experience the same shift when plotted versus density, such that the PF (and its maximum) does not change, as also shown and further discussed in the Supporting Information.

An interesting observation is the trend of the Seebeck coefficient at high *E*_F_ for the heavy aligned band case (inset of Figure 1b). The fully aligned red line is the highest, whereas for the misaligned cases the Seebeck is lower. A crossover in the cases of Δ*E* = 0.2 eV (black line) and Δ*E* = 0.1 eV (blue line) is observed, which can be explained as follows: Starting from the misaligned case (black line) the Seebeck coefficient is low. There is a noticeable reduction at *E* = 0.2 eV, where the upper band is met. The reason is that the upper band is of high mass and low conductivity, with states of reduced current capabilities. Thus, the average energy of the current flow (which defines the Seebeck coefficient–see discussions in the Supporting Information) is shifted to lower energies and the Seebeck is reduced, even becoming negative for some interval (indicating that transport below the Fermi level is stronger). Note that although we consider electrons with negative Seebeck, in all cases we consider in this work we flip the sign of the Seebeck coefficient for convenience. Similarly in the case of the blue line with partial alignment, this deep is shifted at the energy where the upper band is encountered at *E* = 0.1 eV. At higher energies the Seebeck coefficient of the two misaligned cases will tend to approach that of the fully aligned case, and thus the crossover of the two misaligned cases. The fully aligned band case has the highest Seebeck coefficient, as the average energy of the current flow is not hindered by scattering from heavier upper valleys. Notice that, clearly, the Seebeck coefficients follows the expected inverse trend compared to the conductivity.

## 3 Band Alignment with Single-Valley Bands

Typically, the complex electronic structure in materials consists of several bands and valleys, which can differ arbitrarily in energy, and consist of arbitrarily different effective masses. The scattering processes are also complex in general, with different scattering strengths (and deformation potentials) determining the intraband/valley processes for each band, and the interband processes, which can be of different strength as well.^34,53^ Thus, the parameter space for exploration of when band alignment is beneficial is large.

To simplify our investigation and build a first order understanding of the influence of band alignment on the TE properties, we begin by studying a simple system of two single-valley bands, separated by energy Δ*E*. We then assume a common intraband deformation potential, *D*_intra_, but allow for different interband deformation potential, *D*_inter_. This will allow us to focus on the effect of intra- versus interband scattering, i.e., control the degree of scattering as bands are aligned. We consider a base band (“B”) of effective mass  and a band that we bring into alignment (referred to as the aligned band – “A”), for which its mass  is either light or heavy. Distinguishing between the *’light band alignment’* versus the *’heavy band alignment’* cases is also essential in identifying promising design cases, as indicated in Figure 1. In the investigations from here on, for each considered case, we compute the TE power factor as a function of the reduced Fermi level η_F_ = *E*_F_ – *E*_C_ as in Figure 1, and extract the peak of that PF. We then plot that peak value as a function of the energy separation between the bands, Δ*E*, and for the parameters under investigation (*D*_intra_, *D*_inter_, , ).

### 3.1 Intra- versus Interband Scattering for Light and Heavy Band Alignment

**Figure 2:**
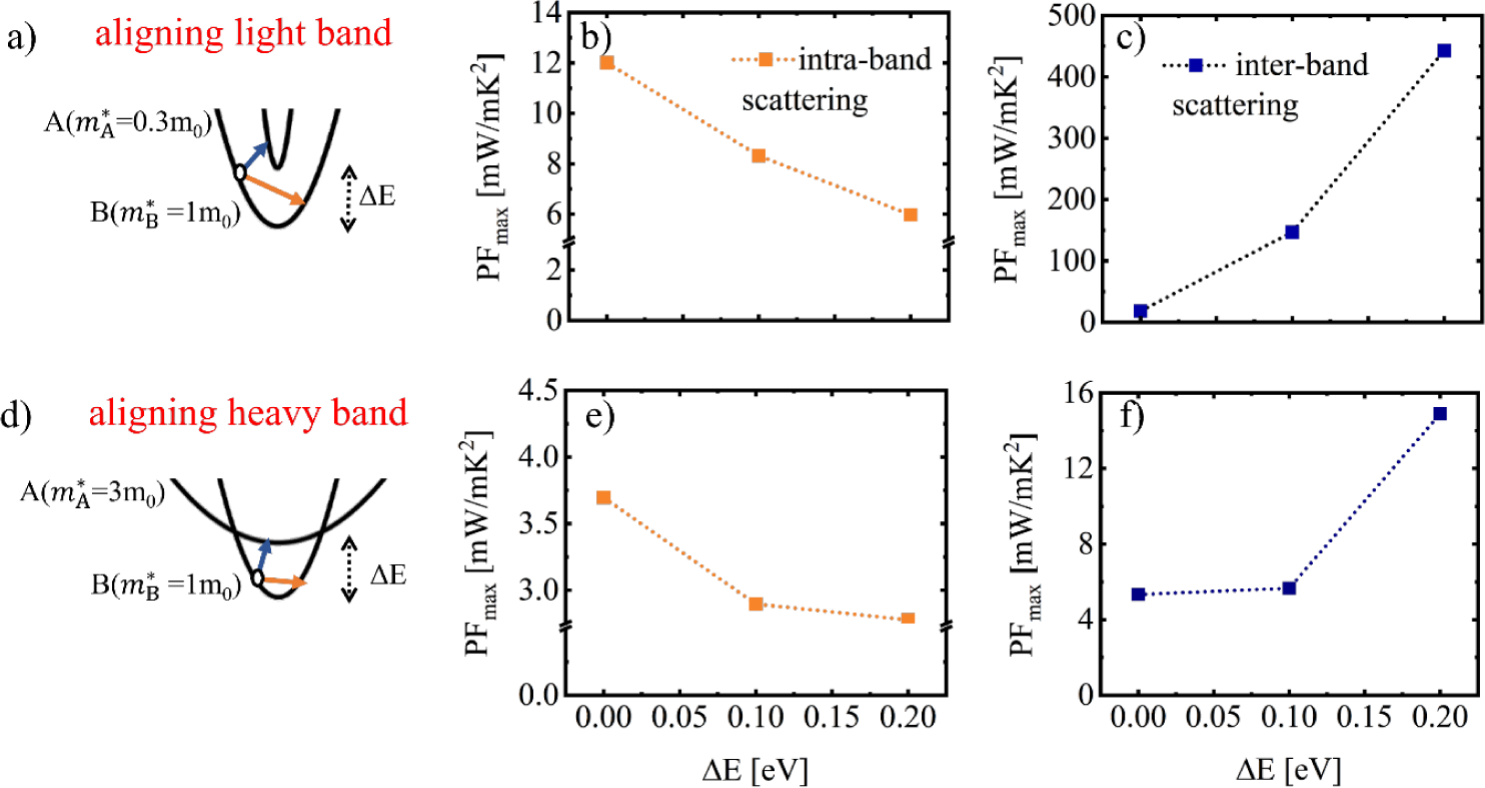
Upper row (a–c): the case for light band alignment. Lower row (d–f): the case for heavy band alignment. First column (a,d: illustrations of the intraband (orange arrows) and interband (blue arrows) scattering processes in the bandstructures. Middle column (b,e): maximum power factors for intraband scattering for the two cases. Third column (c,f): maximum power factors for interband scattering for the two cases.

As a first study we fix and set equal values of *D*_intra_ and *D*_inter_ and focus on the effect of aligning bands of different masses, as well as the effect of intra- versus interband scattering between the base and aligned bands. The schematics in the left column of Figure 2 (Figure 2a,d) illustrate the distinct cases considered. In Figure 2a–c we consider aligning a light band to the base band. Here we use  for the aligned band and keep  as a reference for the base band. The middle column (Figure 2b) shows the peak PF in the case where only intraband scattering is considered, as indicated by the orange transition arrows in Figure 2a. The PF is improved even up to 100% in this case, as Δ*E* is reduced and the bands are aligned. This is expected, since a new, highly conducting band is now additionally contributing to transport, and the scattering rates are unaltered under intraband scattering considerations. In contrast, under solemnly interband scattering conditions, the PF decreases upon band alignment, as shown in Figure 2c. In this case the base valley is highly conducting (since we do not consider intravalley scattering–the reason for the ultrahigh PF values), and bringing another valley close-by introduces scattering for both, and reduces the conductivity and PF. Upon heavy valley band alignment, as shown in the second row, Figure 2d–f, similar observations are encountered. Namely, under intraband only conditions, improvements to the PF are realized, however, these are smaller compared to light band alignment (∼20%), since the heavy band offers reduced conductivity. Under interband only conditions the PF reduces in this case as well, since aligning increases scattering for both bands.

These results illustrate that to realize PF improvements upon band alignment, intraband scattering needs to dominate over the interband scattering processes, while benefits are more pronounced when a light band is brought into alignment. In general, intraband processes are encountered when electrons scatter with acoustic phonons, anisotropic scattering mechanisms such as polar optical phonons and ionized impurities, and other static impurities like defects, boundaries, and alloy scattering.^52^ Intervalley processes, on the other hand, are typically dominated by large wave-vector optical phonon scattering. In typical TE materials the former are strong, thus it is more probable that intravalley processes dominate, but only in the cases where the valleys of the different bands are placed farther away from each other in the Brillouin zone.^11,46,50,52,56,57^

### 3.2 The Degree of Intra- Compared to Interband Scattering

In practice, independent intra- or interband scattering is not the typical case, but the study in Figure 2 reflects the band alignment expectations upon these limiting conditions. In the typical scenario, both processes are present simultaneously, thus, below we quantify the degree upon which the intraversus interband scattering strength ratio allows for PF improvements. For this, we vary the ratio of the deformation potentials which dictate the strength of each of the processes,  from 2 (closer to intraband scattering dominance), to 1, and then down to 0.5 (closer to mimicking interband scattering dominance).

**Figure 3:**
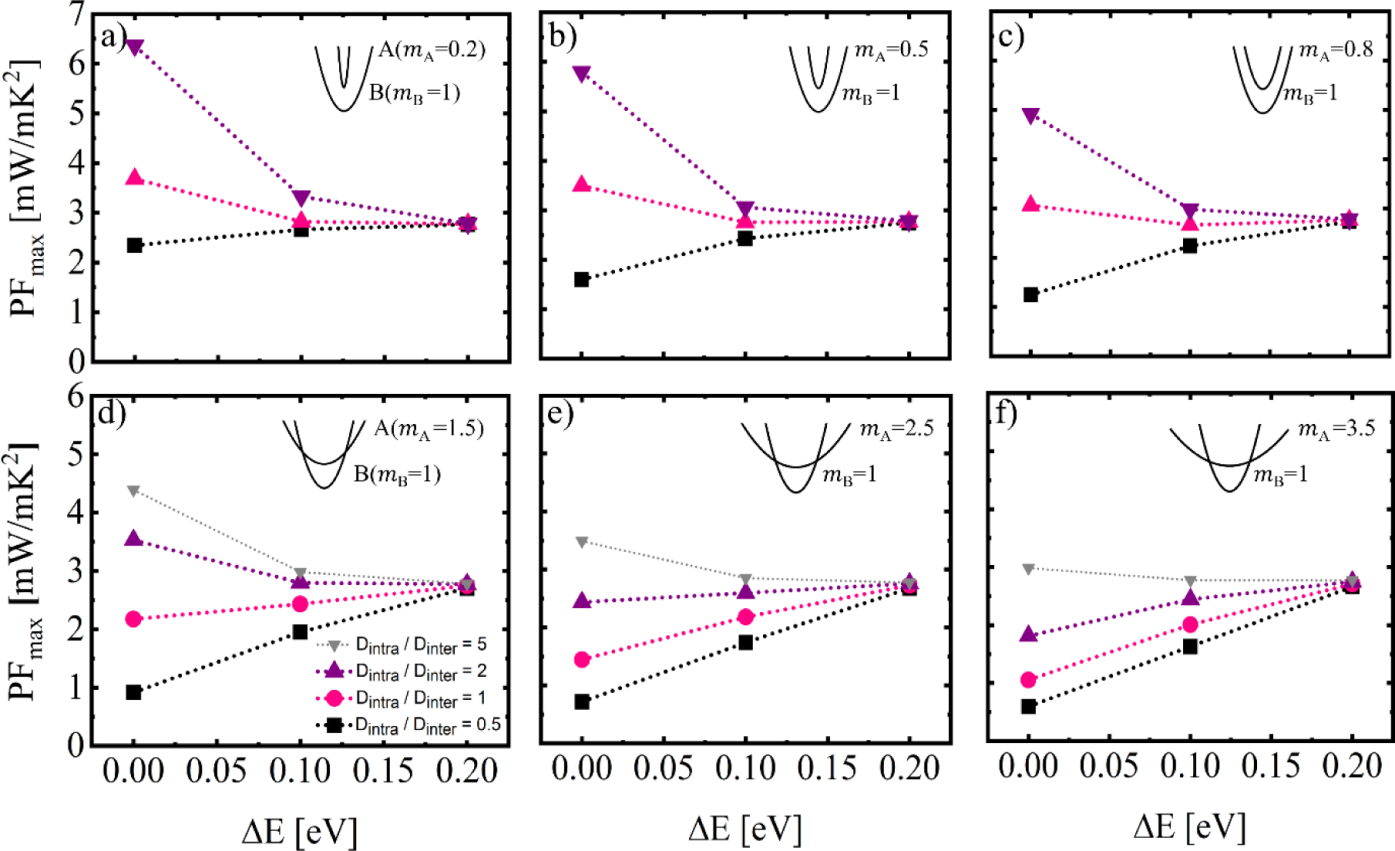
Maximum power factor upon band alignment of two single-valley bands versus their energy difference during the alignment process, Δ*E*. The base band effective mass is set to  in all cases, while the effective mass of the aligned band  is noted in each subfigure in units of the free electron mass m_0_. First row, (a–c): the case for a light aligned band. Second row (d–f): The case for a heavy aligned band. Sets of data for different intra- versus interband scattering cases  are shown. The PF improves upon full band alignment for larger intraband scattering and lighter aligned bands.

We again start with the case of light band alignment, but we also consider different mass ratios, as shown in Figure 3a–c, from  (ultralight) to  (closer to the base band mass of ). In the case of the lighter aligned band mass, Figure 3a, for the larger intra- versus interband deformation potential ratio (, purple line) a ∼100% PF improvement upon full band alignment can be reached, as this also resembles the case in Figure 2b for intraband scattering dominance. As  is reduced, and the situation moves closer to interband scattering dominance, the PF improvement is reduced as well. At equal deformation potentials the improvement drops to ∼30%, whereas for  the PF is slightly degraded upon band alignment (black line) where strong interband scattering is highly supported by optical phonons. As the aligned band effective mass increases (Figure 3b,c), but remains lower than the base band mass, the PF improvements at a specific  and Δ*E* are reduced, while the degradation when interband becomes stronger, is larger (black lines). Essentially, the scattering introduced on carriers in the base band into each heavier-and-heavier aligned band, becomes stronger due to the ever-increasing scattering DOS, which limits its conductivity and the PF.

The trend of weakening the PF improvements at a certain  ratio and Δ*E* continues as the aligned band mass, , is increased and overpasses the mass of the base band (). This is shown in Figure 3d–f, where now a heavy band is essentially brought into alignment, here with masses . In this case, band alignment gives marginal PF benefits when the strength of intraband scattering is larger, i.e.,  (purple lines), but these benefits are diminished when the aligned band mass increases further (Figure 3e,f). Here for any lower  values the PF degrades for all masses of aligned bands. However, the PF increases somewhat, or at least does not decrease, upon band alignment for all the heavily aligned bands in limiting case of the presence of ultrastrong intravalley scattering (gray line).

Thus, this analysis shows that upon band alignment, PF benefits can be achieved when the aligned band mass is lighter than the base band mass. Even so, benefits are still reached when the relative scattering strength of the intraband scattering process is of the order of, or stronger than the interband scattering process (as denoted here by the deformation potentials which determine the scattering process, i.e.,  ≳ 1). As the mass of the aligned band becomes heavier, a much larger ratio is needed to realize benefits, which can be unrealistic in typical materials.

**Figure 4:**
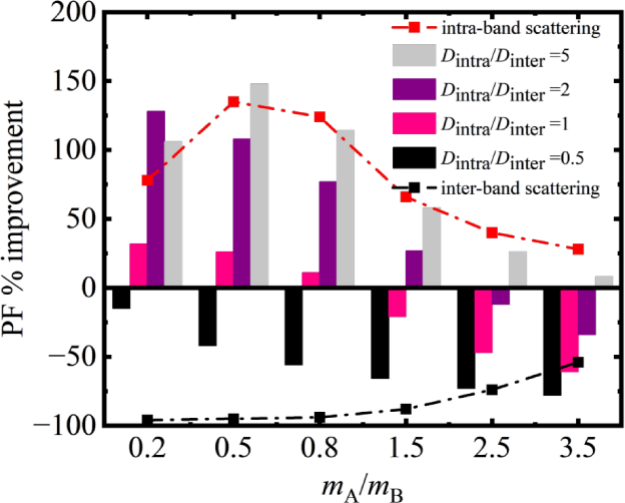
Bar chart illustrating the percentage improvement in the maximum PF upon full band alignment of two single-valley bands versus different effective mass ratios between the aligned and base bands, and for various intra- versus interband scattering strengths as set by the deformation potential ratios of the two processes, . The red and black dashed–dotted lines show the cases of only intra- and only interband scattering, respectively.

A summary of these findings is better illustrated in Figure 4, in which presents a bar-chart of the percentage change of the PF upon full band alignment versus the mass ratio of the aligned and base band for the different  ratios. For the large, aligned band effective mass  region (right side), most of the bars are negative, indicating reduction in the PF upon band alignment. As the aligned mass becomes smaller than the base band mass, only then the bars become positive and increase as that mass is reduced even further; and noticeably only for the cases where the intraband processes are stronger compared to the interband scattering processes (purple and pink bars).

The red dashed-dot line and the square symbols indicate the case where *only intraband scattering* is considered, i.e., the envelope of the best case scenario for the performance improvement upon band alignment. Note that as the mass of the aligned band is reduced to values smaller than  (left, upper part of Figure 4), we reach a point of diminishing returns, where the *PF improvement* upon band alignment starts to reduce. The absolute PF value is still larger as shown in the Supporting Information, Figure S.1. However, since the much lighter aligned band contributes significantly to transport and the PF to begin with, aligning it with the heavier base band increases its scattering and offers less of an improvement. Interestingly, in this case, some intervalley scattering can be beneficial to providing relative PF improvements, as in that case the lighter aligned band becomes more resistive and contributes to transport less to begin with (although the absolute value of the PF is reduced in this case).

With the black dashed-dot line we show the case where *only interband scattering* is considered, in this case it is the envelope for the worst performance improvements. This line does not follow the trend of the negative value bars, because those are not in the only interband scattering regime. The reason for this deviation is that for very low effective masses of the aligned band, that aligned band dominates the PF, which then is drastically reduced once it is brought in the vicinity of the base band which introduces scattering (similar to what we describe earlier in Figure 2c).

An important point is which of the two quantities controls the behavior we show in Figures 2–4. As also shown and discussed for the case of Figure 1, the conductivity and changes to that upon band alignment, is what dominates the changes in the PF, while the Seebeck coefficient has a smaller influence. In the Supporting Information (Figures S5.1 and S5.2) we show a large set of data for the TE coefficients in Figures 2–4, plotted versus the Fermi level. The fact that the conductivity determines the PF performance by a large degree is evident both in the case of light and heavy aligned bands, but also both in the cases of stronger intraband scattering or stronger interband scattering. This reflects directly to our main observation, that light aligned bands of higher conductivity are what allow for higher PF improvements, rather than heavier aligned bands that offer higher density of states (DOS) but lower conductivity. Note that in our simulations we kept the base band constant and altered the aligned band. What matters finally is the ratio of the two masses, thus we could have altered the base mass and kept the aligned band mass the same, without changing our conclusions.

The above analysis is performed for the simple case where a single degenerate band is aligned with another single degenerate band. In practice, typical materials involve bands at high symmetry points of the Brillouin zone with multiple band degeneracies. For example, the L valley of 8 half valleys (*N*_v_ = 4), or the W valley of 24 one-third valleys (*N*_v_ = 8). Thus, in practice we are faced with the possibility of aligning single, or multivalley bands upon either single or multivalley bands. We have identified that when the aligned band is heavier, and interband scattering dominates, benefits cannot be achieved. However, it is interesting to examine if this will still be the case if multivalley bands are aligned, in which case multiple conduction channels are added to the transport energy window. This is discussed in the section below.

## 4 Band Alignment with Multivalley Bands

**Figure 5:**
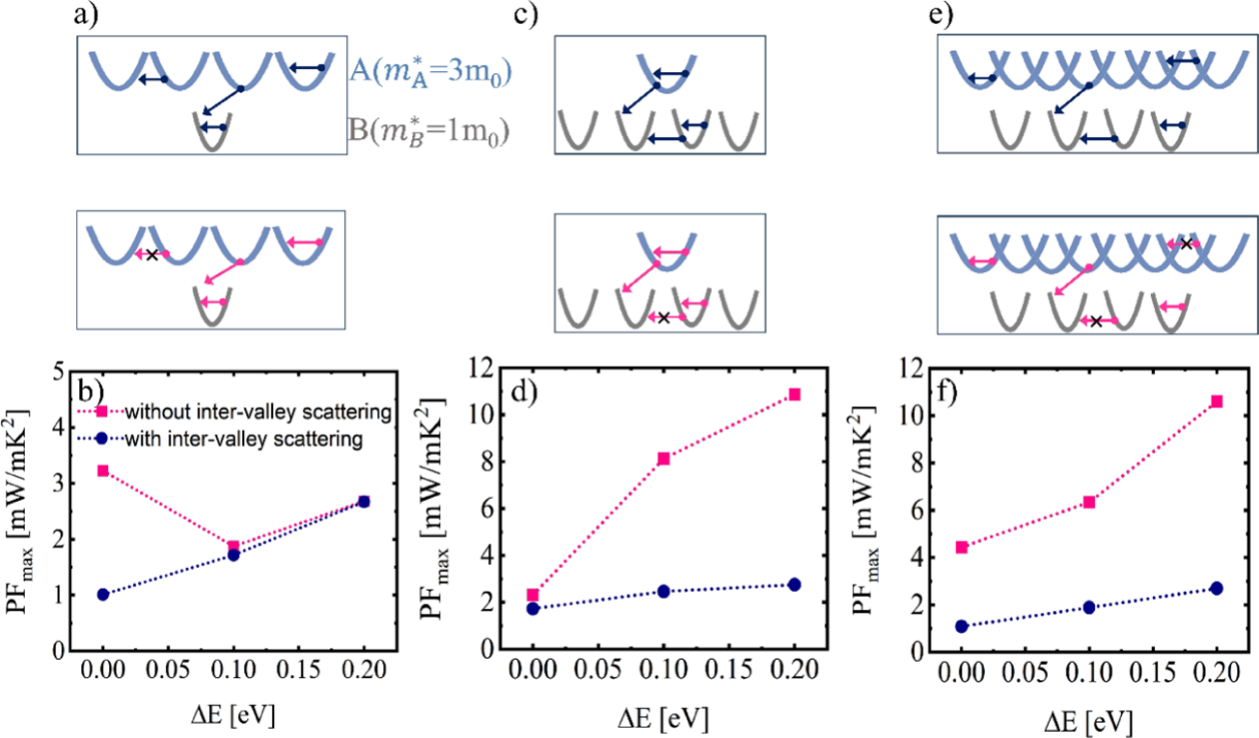
Maximum PF change upon band alignment of a band with heavier effective mass valleys compared to those of base band. Three different cases are shown as indicated by the schematics in the top rows: (a,b) a band with multiple heavy valleys is aligned with a lighter-valley base band. (c,d) A single-valley heavy band is aligned with a band of multiple lighter valleys. (e,f) A band with multiple heavier valleys is aligned with a band with multiple lighter valleys. The aligned band has valleys with  , whereas the base band has valleys with  (curvature not drawn to scale). Intravalley and interband scattering are broadly considered. For each bandstructure two scattering cases are considered in addition: intervalley scattering between valleys of the same band (blue lines), and the absence of such intervalley scattering (magenta lines).

The schematics on the top panel of Figure 5 illustrate the different scattering situations we consider. In all cases we consider the alignment of heavy effective mass bands, i.e., . Since this was the case where it was most difficult to provide PF improvements, we seek to examine if there is a possibility to reverse this by aligning multiple bands. Here we consider intravalley scattering for both base and aligned bands and interband scattering between them, but in addition we distinguish between two different scattering cases for the valleys of the same band: (i) the case where intervalley scattering is included (as indicated by the arrows in the top schematic), and (ii) the case where intervalley scattering is excluded (indicated by the magenta arrows in the lower row schematics). The latter is the case where ODP is weak, or the different valleys of the same band are placed far from each other in the Brillouin zone, and/or possibly IIS or POP is stronger, such that intervalley scattering is the weaker of the scattering processes. In the former the reverse can be true. For simplicity we consider the same deformation potential across all scattering events, and same mass for the aligned band valleys , a mass that was previously detrimental to the PF even at strong intravalley scattering).

The first column (Figure 5a,b) deals with the alignment of a multivalley band with *N*_v_ = 4, onto a single valley base band (*N*_v_ = 1). This could be the case with TE materials such as half-Heusler (HH) alloys i.e., p-type TiCoSb, and TiNiSn^58,59^ which have multivalley heavy bands at L and a single valley band at the Γ point in the Brillouin zone (as shown in Figure S2.1 of the Supporting Information).

In the first scattering consideration case, where intervalley scattering is allowed within the same band, band alignment degrades the PF performance (blue line in Figure 5b). The reason is that since carriers from each valley scatter into each other, the contribution of that aligned band to transport is similar as that of a single band (more valleys, but more scattering). In addition, the interband scattering from the aligned to the base band that is present, degrades the PF performance of the base band. Thus, the PF is overall degraded. In the second scattering case, where we exclude intervalley scattering between the multivalleys of the aligned band, the situation is slightly better for the PF upon band alignment (magenta line in Figure 5b). Initially interband scattering for the lower energy band increases by 4-fold (since ) as the aligned band is lowered in energy, and the PF is lowered. This reduction is mitigated at full band alignment by the fact that four more valleys now contribute to transport. Still, however, even in this case, PF improvements are not observed, and at best the PF of the fully aligned bandstructure remains the same as the one of the fully nonaligned one.

Thus, aligning multiple (even heavy) valleys onto a single base valley might seem as a tempting favorable scenario, but it can be beneficial only when the combined system of those aligned valleys is more conductive compared to the base valley. For this, a large valley degeneracy is needed, but also there has to be reduced scattering within those aligned valleys themselves, i.e., reduced or no intervalley scattering. This allows the high valley degeneracy to provide a highly conducting aligned system, despite their heavy mass. If intervalley scattering is present, however, then the enhanced scattering within that aligned system reduces its conductivity and makes the situation similar to the alignment of a single heavy band, which as we discussed in the first section of the paper, it is not beneficial to the PF. We assume intraband scattering is present in Figure 5a,b. If intraband scattering is strong, then the contribution to the PF of the base band is reduced due to scattering from the base band into the heavy valleys, thus this mitigates the PF improvements that can be reached. If intraband scattering is not present, however, then the situation is beneficial to the PF as discussed in the first part of the paper. Essentially the base band conductivity remains intact, and additional conductivity from the heavy valley band system is brought into the picture, which increases the PF.

In the second case (middle column, Figure 5c,d), we consider the reverse example, i.e., the alignment of a single-valley band (for example one that is located at the Γ point of the BZ), while the base band is now considered to consist of multivalleys with  (at L). This could resemble materials such as p-type ZrCoBi, and ZrCoSb^60^ where single-valley may be aligned with multivalley band as shown in the Supporting Information, Figure S2.2. Here again, we consider intravalley scattering for all valleys and intraband scattering, and also as before: (i) the case where intervalley scattering is present between the base valleys, and (ii) when intervalley scattering is absent, as indicated by the arrows in the schematics on the top panel (Figure 5c). In this case the four base valleys dominate transport to begin with. In the first case (blue line in Figure 5d), intervalley scattering keeps the PF low.^32^ Aligning a heavier valley which introduces additional scattering, only degrades the PF more. In the second case, removing the intervalley scattering in the base valleys to improve transport increases the amplitude of the PF to begin with (magenta line in Figure 5d). However, upon full band alignment, the PF is reduced significantly due to the large increase in scattering that the large DOS of that aligned band introduces. Thus, band alignment does not help in this situation either.

In the third case (Figure 5e,f), we consider aligning a multivalley band ( , e.g., at the W point in BZ) to a multivalley base band (, e.g., at the L point) as well. In general, the PF magnitude is greater in such cases, since many valleys offer more conduction channels, as also shown in a prior work.^61^ Band alignment with these degeneracies may be realized in materials such as p-type NbFeSb, VFeSb and AgSbPbSnGeTe_5_ as shown in the Supporting Information, Figure S2.3.^62,63^ For example, the last case, high entropy material, gives high TE efficiency due to band convergence of four multiple valley bands i.e., L, W, Γ, and X. In such situations, the presence of a multivalley base band provides a strong contribution in determining the PF to begin with, similar to the previous case. Thus, the maximum value of PF is observed before alignment (Δ*E* = 0.2 eV), also in agreement with a previous study.^48^ In either case, in the presence of intervalley scattering or its absence, the PF is reduced upon band alignment as shown in Figure 5f (although the PFs are always larger as expected when intervalley scattering is absent). This is because the weighted contribution to transport from the many heavy-band valleys is less in this case, since the base band also has multiple valleys, and the introduction of interband scattering degrades the PF.

Thus, the overall conclusion from this section, is that aligning heavier effective mass bands compared to the base band effective mass does not offer advantages to the PF in the presence of intra/inter-band scattering, even if the aligned band consists of multiple valleys for transport, and even if intervalley scattering within the same band is absent to increase the band’s conductivity. At best, upon full band alignment, the PF retains its original value. One condition that can improve the PF upon full band alignment of a heavy band, is if that aligned band is highly conducting, for example if both the intra- and intervalley scattering processes within that aligned band are weak, and degeneracy is high. This can be the case of aligning a multivalley heavy band upon a single-valley base band as shown in Figure S3 of the Supporting Information. Then although upon band alignment the base band will experience increased scattering, the contribution of the highly conducting aligned band to transport can be significant, and the overall PF could increase. Such highly conducting high energy bands (compared to the lower energy base bands), however, would not be easily encountered in realistic material cases.

**Figure 6:**
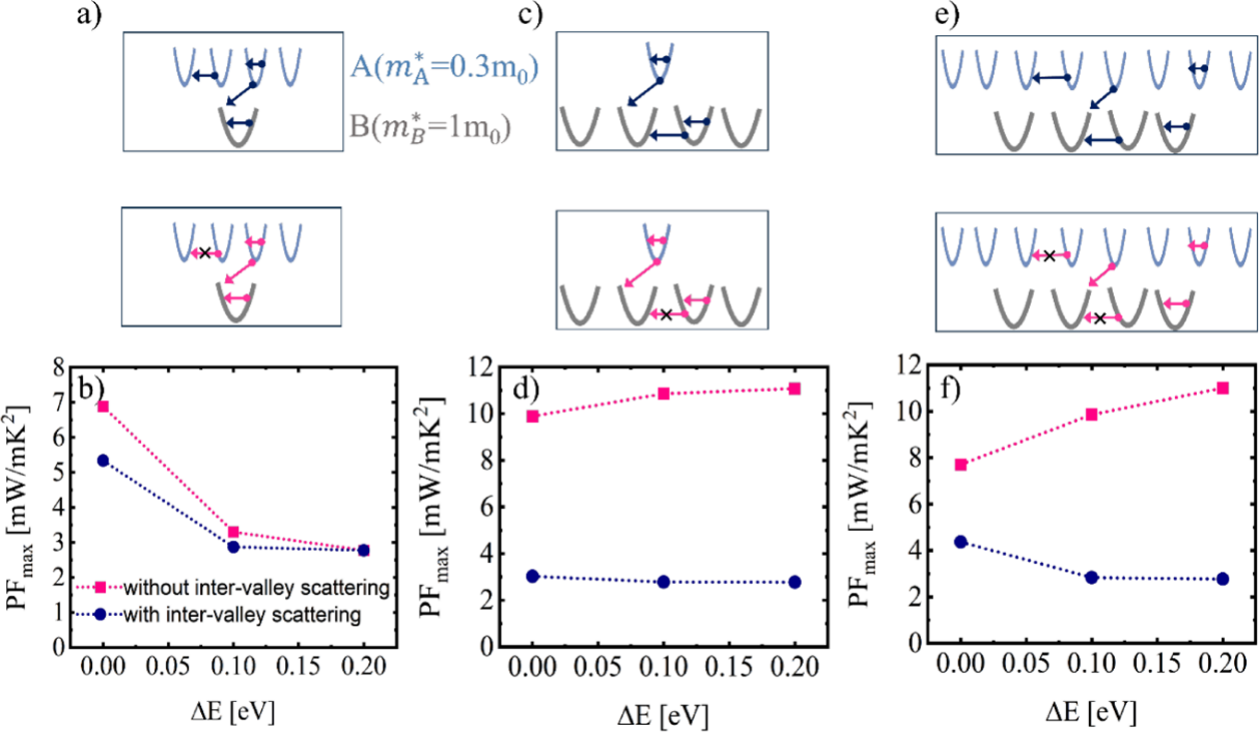
Maximum PF change upon band alignment of band with lighter effective mass valleys compared to those of base band. Three different cases are shown as indicated by the schematics in the top row: (a,b) a band with multiple light valleys is aligned with a single-valley heavier mass base band; (c,d) a single light valley band is aligned with a base band with multiple heavier valleys. (e,f) a band with multiple light valleys is aligned with a multiple-valley heavier band. The aligned band has valleys with  whereas the base band has valleys with  (curvature not drawn to scale). Intravalley and interband scatterings are broadly considered. For each bandstructure two scattering cases are considered in addition: intervalley scattering between valleys of the same band (blue lines), and the absence of such intervalley scattering (magenta lines).

The situation for the PF is of course subject to change for lighter aligned band masses  , and if interband scattering weakens on top of that, such that the aligned bands become more conductive this way. Such cases are in favor of PF improvements, as discussed in the previous section. Namely, the more the lighter valleys that are brought into alignment with respect to the number of heavier base valleys, the more the PF improvement. This is shown in Figure 6, which again considers intravalley and interband scattering, and the two cases of: (i) intervalley scattering (blue arrows in the top panel schematics), or (ii) the absence of intervalley scattering (magenta arrows in lower panel schematics). When multiple light bands are aligned on top of a single base band, PF improvements are reached (Figure 6a,b). In this case the four additional bands bring almost four times the improvement the single band presented earlier (Figure 1c), proving that valley degeneracy is an important parameter for high thermoelectric performance under these conditions.^46^ If a single light-valley band (Figure 6c,d) or a multiple light-valley band (Figure 6e,f), is aligned on top of multiple heavy bands, no PF improvements are reached (magenta lines). This is because a multivalley heavy base band offers a large interband scattering DOS as compared to single or multiple light aligned bands, in such a way, such that the aligned valleys cannot challenge the transport dominance of the multiple-valley heavy base bands. Only a moderate PF improvement can be reached in both cases, and this only when the conductivity of the base heavy band is already weakened by intervalley scattering (blue line). Thus, overall, even in this case of lighter aligned bands, the aligned band needs to provide significant conductance benefits over the base heavier band valleys for improvements to be achieved. PF improvements can be reached in this case, by even up to 100% and beyond, i.e., more than doubling the PF, but as in most case described above when: (i) intraband scattering dominates over interband scattering, (smaller improvements are obtained when interband scattering exists, even down to the level when intra- and interband scattering deformation potentials are comparable to each other , and (ii) when aligning highly conducting (degenerate) valleys for which not only interband scattering is weak (between the aligned band and the base band), but also when intervalley scattering is also weak (between the degenerate valleys themselves).

## 5 Conclusion

In this work, we used a multiband parabolic effective mass model and the Boltzmann Transport Equation to investigate and quantify the effect of band alignment on the thermoelectric power factor of multiband/valley electronic structure materials. We have considered different band alignment conditions that can improve the power factor in the presence of combinations of intra- and interband/valley scattering. We show that in general, upon band alignment, power factor improvements can be reached when the aligned band is highly conducting with respect to the base band, and it does not interact strongly in terms of interband scattering between them. Specifically, this can be achieved when intraband scattering dominates over interband scattering, and primarily when a lighter valley band is brought into alignment, while smaller improvements are realized when a heavy valley band is brought into alignment. In practical terms this can reach around 100% improvements.

In the presence of dominating interband scattering, power factor improvements cannot in general be achieved, especially when heavy-valley bands are brought into alignment. Even when the heavy-valley aligned band consists of multiple degenerate valleys, benefits still cannot be realized unless that heavy-valley band is (unrealistically) highly conducting with limited intra- and intervalley scattering between itself and it is not interacting strongly with the base band. Note, however, that many promising thermoelectric materials are polar, and ionized impurity scattering is strong under high doping conditions. These are both anisotropic scattering mechanisms which favor intravalley scattering. In these materials the dominance of intravalley scattering can allow power factor benefits upon-band alignment. Our findings would help to correctly and easily identify promising material candidates to focus band alignment studies on, which would expedite the realization of high-performance thermoelectric materials.

## Supporting Information Available

Power factor values for the data in Figure 4 of the main paper (Section S1); examples of real material bandstructures with misaligned bands (Section S2); heavy band alignment upon only interband/valley scattering considerations (Section S3); Seebeck coefficient shape explanations (Section S4); full data for Figures 2 and 3 of the main paper (Section S5); results on altering the base band effective mass (Section S6) (PDF)
