# Supplementary material for: Conditions for Thermoelectric Power Factor Improvements upon Band Alignment in Complex Bandstructure Materials
Source: ACS Appl Energy Mater. 2025 Jan 24;8(3):1609–19. doi: 10.1021/acsaem.4c02747 (PMC11815832; doi:10.1021/acsaem.4c02747)
Supplement: Supplementary file 1 — ae4c02747_si_001.pdf [file ae4c02747_si_001.pdf]

Supporting Information

Conditions for thermoelectric power factor improvements upon band alignment in complex bandstructure materials

Saff E Awal Akhtar[+] and Neophytos Neophytou[*]

[+]saff-e-awal.akhtar@warwick.ac.uk, [*]N.Neophytou@warwick.ac.uk

Section 1: Power factor values for the data in Fig. 4 of the main paper

The figure below presents the power factor (PF) for the data presented in Fig. 4 of the main paper, i.e. as the mass of the aligned band changes from a light mass (left side, $m_A^* < 1m_0$) to a heavy mass (right side, $m_A^* > 1m_0$)) while the base band has $m_B^* = 1m_0$. The figure shows the actual PF values, but with positive bars it shows the power factor cases that result in improvement of the PF upon band alignment, while with negative bars indicates the cases where full alignment results in PF degradation. The actual PF values are the absolute values.

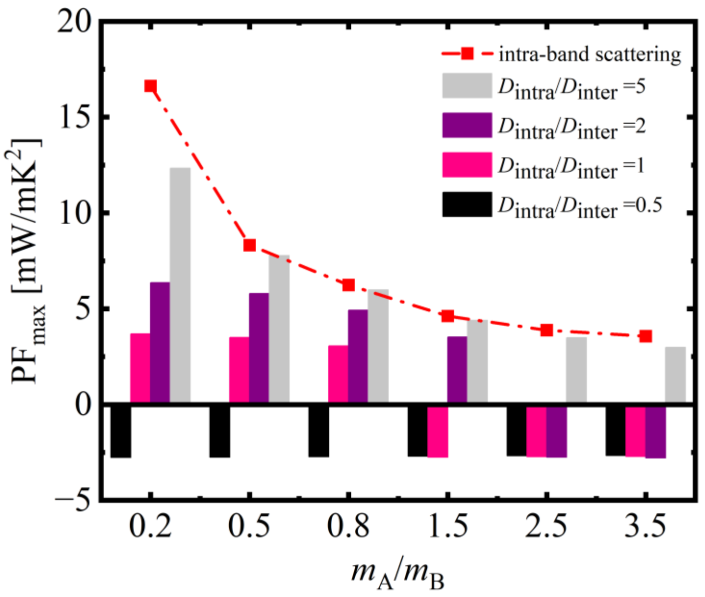

Figure S1.1: Bar chart illustrating the maximum PF during the band alignment process for the cases described in Fig. 4 of the main text. The aligned/base bands are initially separated by $\Delta E = 0.2$ eV, while upon alignment they have $\Delta E=0$ eV. Values for different effective mass ratios are shown between the aligned (m_A^*) and base (m_B^*) bands. These calculations have been performed for various intra- versus inter-band scattering strengths as set by the deformation potential ratios of the two processes, $\frac{D_{intra}}{D_{inter}}$ and noted in the figure. The red dashed-dotted line shows the cases of only intra-band scattering considerations.

Section 2: Examples of material bandstructures with misaligned bands

Here we show the relevant bandstructures of half Heusler thermoelectric materials indicating the possibility of band alignment in the valence band for TiCoSb, TiNiSn (Fig. S2.1-aligning many valleys from L onto a single Γ valley), ZrCoBi, and ZrCoSb (Fig. S2.2-aligning a single Γ valley onto multiple L bands), and VFeSb, NbFeSb (Fig. S2.3-aligning mutliple W bands onto multiple L bands). The dots show the band extrema. The red arrows show the possible bands to be aligned with the base valence band. ΔE(eV) in each figure represents the energy offset between the base and aligned bands in the valence band.

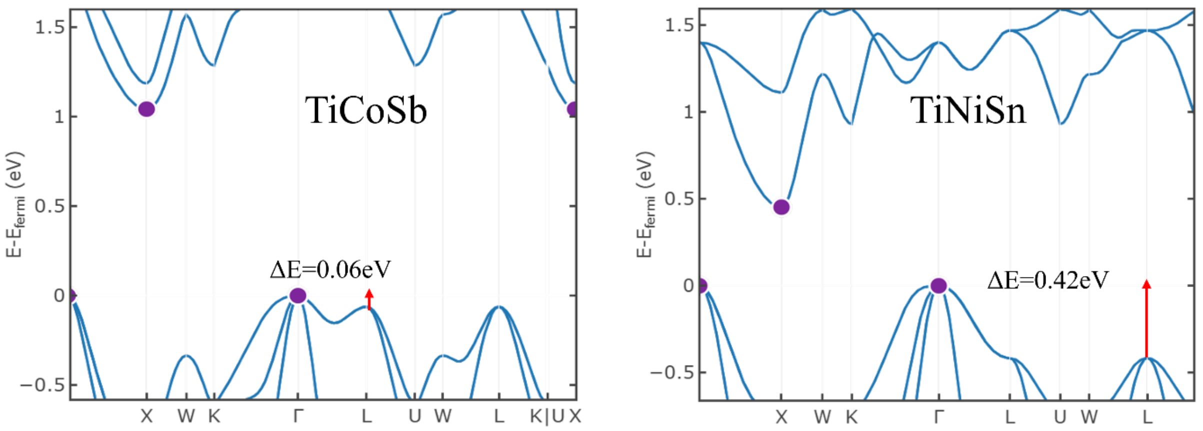

Figure S2.1: The bandstructures of (a) TiCoSb [1], and (b) TiNiSn [1] around the bandgap. These are examples of multi-valley band (at L) alignment upon single-valley band (at Γ).

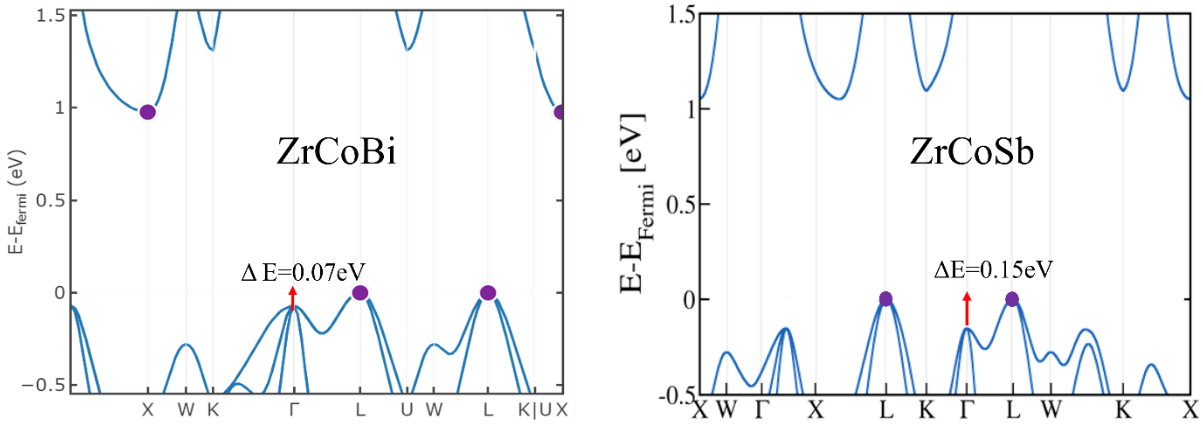

Figure S2.2: The bandstructures of (a) ZrCoBi [1], and (b) ZrCoSb [self-calculated] around the bandgap. These are examples of single-valley band (at Γ) alignment upon multi-valley band (at L).

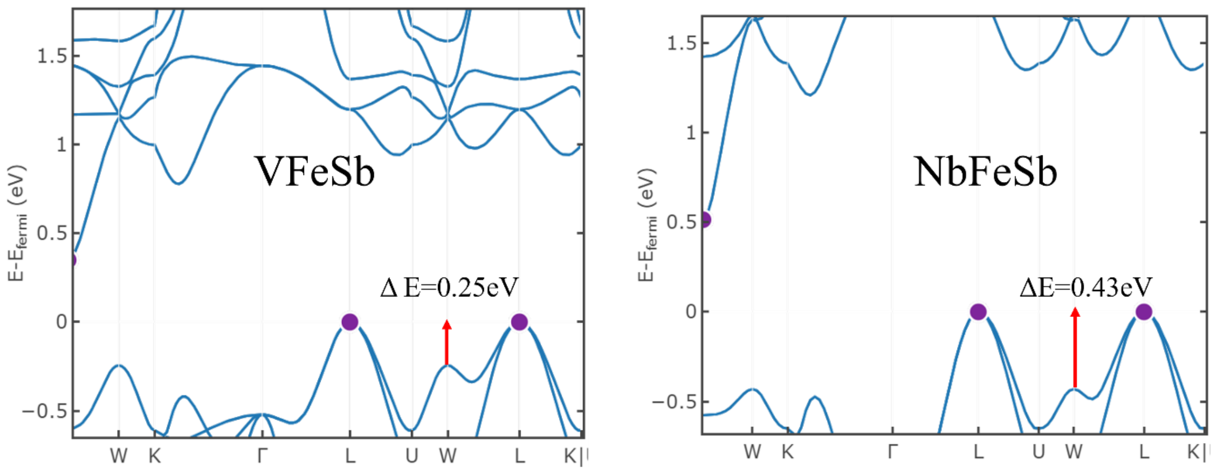

Figure S2.3: The bandstructures of (a) VFeSb [1], and (b) NbFeSb [1] around the bandgap. These are examples of multi-valley band (at W) alignment upon multi-valley band (at L).

Section 3: Heavy band alignment upon only inter-band/valley scattering considerations

Figure S3 shows a schematic illustration of inter-band/valley scattering only (exclude intra-band/valley scattering) for multi-valley heavy band alignment upon a single band. When only inter-band scattering is present (without inter-valley), then power factor benefit is achieved upon full band alignment (green arrows and lines). In the case where inter-valley scattering is present, i.e. the aligned bands become now more resistive, PF degradation is experienced upon full band alignment.

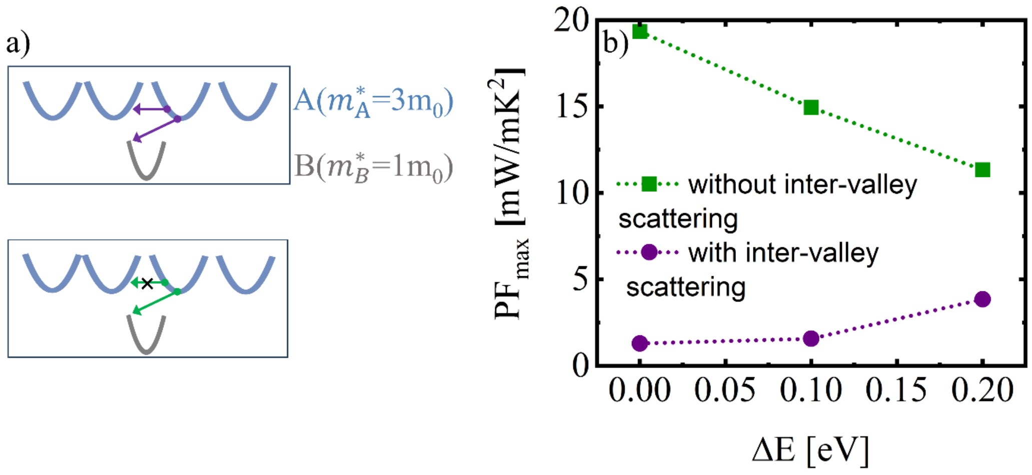

Figure S3: Maximum PF for two cases upon band alignment. Heavy band alignment between aligned band A (m_A^*=3) with valley degeneracy N_v^A = 4, and base band B (m_B^*=1). With green we show the case where only inter-band/valley scattering is present, whereas with purple we also consider inter-valley scattering between the valleys of multi-valley bands as well (see arrows in band illustrations). In both cases intra-valley scattering is omitted.

Section 4: Seebeck coefficient shape explanations

Figure S4.1 shows the Seebeck coefficients from Fig. 1 of the main paper in zoomed-in versions (same as the insets of Fig. 1) to illustrate how they change in the higher Fermi level regions where the PF peaks, upon bringing in another valley. Changes in the Seebeck coefficient upon band alignment are typically hidden when we plot the Seebeck coefficient from different scenarios in terms of the Fermi level, E_F. These changes are much more pronounced if the Seebeck coefficients are plotted versus density, n. Thus, we also present the figures for S plotted versus density, where the changes are more pronounced and follow the usual trend of increasing Seebeck with increasing density of states, DOS.

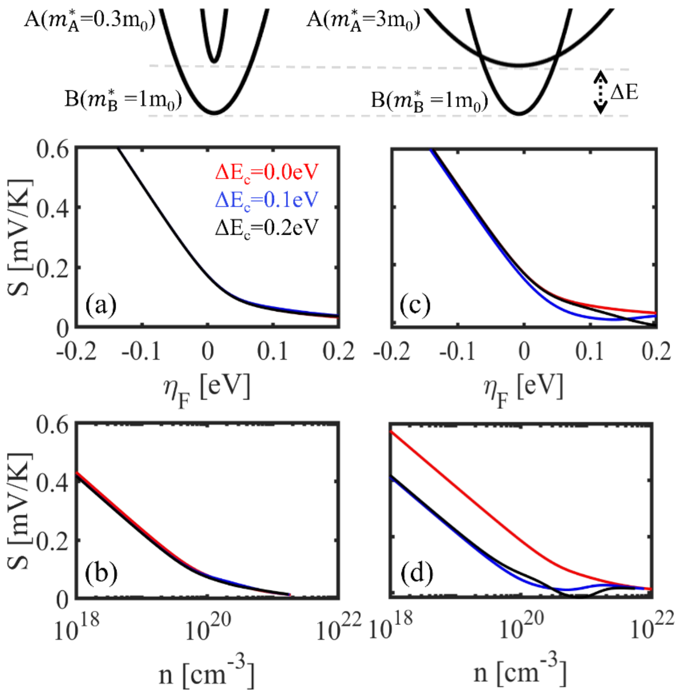

Figure S4.1: The Seebeck coefficients from Fig. 1 of the main paper in zoomed-in versions plotted versus Fermi level (middle column), and versus density (third column).

To explain the almost negligible shift in S when plotted versus the Fermi level, we start with the definition of the Seebeck coefficient as ‘the average energy of the current flow with respect to the Fermi level’. To see this, we can rearrange the original equation for the Seebeck coefficient:

$$S_{(E_F)} = \frac{qk_B}{\sigma_{(E_F)}} \int_E G(E) \left(\frac{E-E_F}{k_B T}\right) \left(-\frac{\partial f}{\partial E}\right) dE \quad (1)$$

where $G(E)=v(E)^2 g(E)\tau(E)$ is the transport distribution function, and $\left(-\frac{\partial f}{\partial E}\right)$ is the energy derivative of Fermi Dirac distribution function, sharply peaked around Fermi level.

We can expand the numerator of Eq. (1) as:

$$\int_E G(E)(E-E_F)\left(-\frac{\partial f}{\partial E}\right) dE = \int_E G(E)(E)\left(-\frac{\partial f}{\partial E}\right) dE - E_F \int_E G(E)\left(-\frac{\partial f}{\partial E}\right) dE \quad (2)$$

Substituting back into the main equation (Eq. 1), we obtain:

$$S_{(E_F)} = \frac{q}{T} \frac{\int_E G(E)(E)\left(-\frac{\partial f}{\partial E}\right) dE - E_F \int_E G(E)\left(-\frac{\partial f}{\partial E}\right) dE}{\sigma_{(E_F)}} \quad (3)$$

and with

$$\sigma_{(E_\mathrm{F})} = \int_E G(E)\left(-\frac{\partial f}{\partial E}\right) dE\ , \tag{4}$$

we obtain:

$$S_{(E_\mathrm{F})} = \frac{q}{T}\ \frac{\int_E G(E)(E)\left(-\frac{\partial f}{\partial E}\right)dE}{\int_E G(E)\left(-\frac{\partial f}{\partial E}\right)dE} - \frac{E_F \int_E G(E)\left(-\frac{\partial f}{\partial E}\right)dE}{\int_E G(E)\left(-\frac{\partial f}{\partial E}\right)dE} \tag{5}$$

Here the first term is the 'weighted energy of conducting carriers that participate in electrical transport, or the so-called 'energy of the current flow', $\langle \mathrm{E} \rangle$, as:

$$\langle \mathrm{E} \rangle = \frac{\int_E G(E)(E)\left(-\frac{\partial f}{\partial E}\right)dE}{\int_E G(E)\left(-\frac{\partial f}{\partial E}\right)dE} \tag{6}$$

Finally we can define the Seebeck coefficient in form of the energy of the current flow, $\langle \mathrm{E} \rangle$, as:

$$S_{(E_\mathrm{F})} = \frac{q}{T}\ [\langle \mathrm{E} \rangle - E_F] \tag{7}$$

Thus, *S* is defined by the energy of the current flow with respect to the Fermi level. We can now imagine the scenario where we align a second valley on the base valley of equal DOS. This will double the DOS at the band edge. If we keep the Fermi level fixed, at the original position (as a gedanken experiment at this point – essentially allowing the density to double), clearly *S* will not change, as the energy of the current flow in each valley independently, and combined, will be the same as the one in the original base valley. This is the case when we plot *S* versus η_F, as in this work. If the mass of the aligned band varies, then some small changes in *S* are expected as the energy of the current flow will be changed slightly, however, not due to the DOS directly, but due to the complexities of transport. In reality, however, if the carrier density in the material remains constant (i.e. as determined by the doping density) – and this can be a more realistic scenario, then when the DOS doubles the Fermi level will shift lower compared to the band edge to retain the carrier density. In this case the Seebeck coefficient increases, since $\langle \mathrm{E} \rangle$ -$\mathrm{E_F}$ increases, essentially shifting the *S* vs *n* curve to the right (see red line in the figure above). Note that this is exactly what happens in the Pisarenko plot when a material acquires a larger DOS upon alloying or other band modifications, at (mostly) unchanged density. Of course, how we plot the Seebeck coefficient does not affect our results, as the conductivity and PF will also shift together with *S*, and the peak PF that the paper examines, will be unaffected. This is shown clearly in the Fig. S4.2 below, which shows that all TE coefficients shift once plotted versus density.

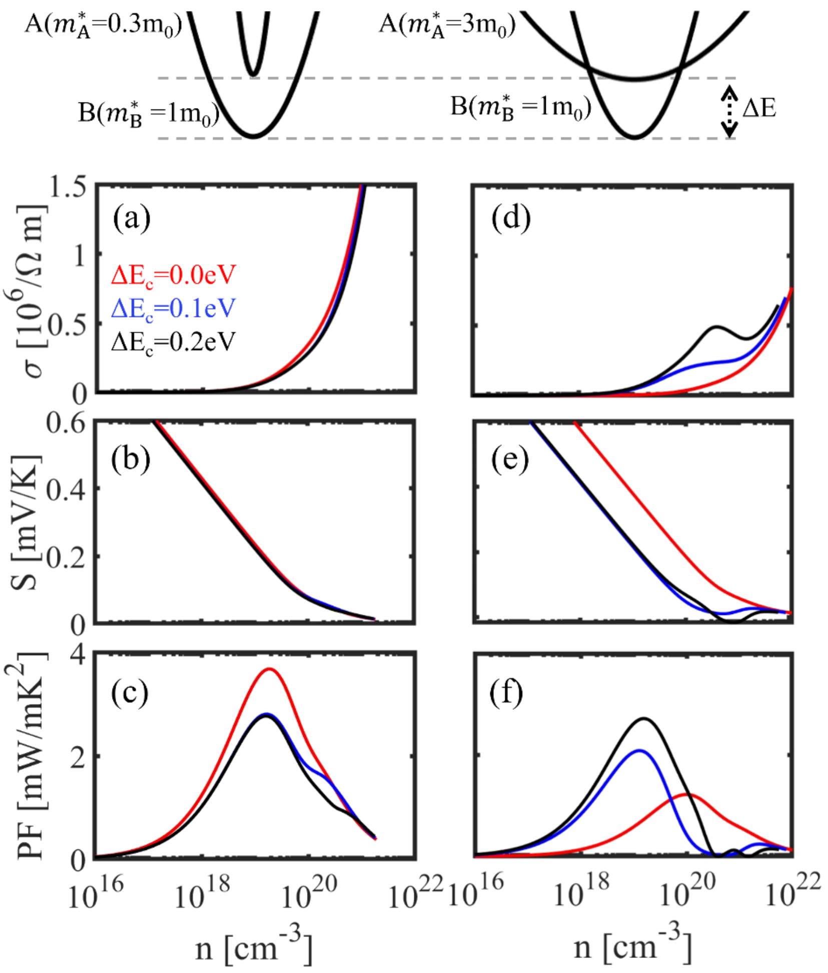

Figure S4.2: The TE coefficients for Fig. 1 of the main manuscript plotted versus density.

Section 5: Full data for Figures 2 and 3 of the main paper

Here we show full data for σ, S and PF for what presented in Figs. 2, 3 and 4 in the main paper. Figure S5. 1 shows in each of the sub-figures two sets of three lines (for ΔE = 0 eV, ΔE = 0.1 eV, and ΔE = 0.2 eV): the first set is for intra-band scattering only (dashed lines) and the second set for inter-band scattering only (solid lines). In the case of a light aligned band (left column), in the intra-band scattering only case (dashed lines) the improvements in the PF originate from improvements in σ, whereas S does not change much, as also explain in the response to the first comment. In the case of inter-band scattering only (solid lines), large σ and PF values are observed in the misaligned case (black solid lines) since intra-band scattering is suppressed, and the conductivity of the lower base band continues to increase until the upper band is reached. Thus, all the TE coefficients are shifted towards higher energies, and the higher conductivity leads to very-high PFs (although this is under the unrealistic assumption of

complete suppressed intra-band scattering). As the bands are aligned, a left shift in the TE coefficients is observed, and a strong reduction in the PF due to increase in inter-band scattering as the upper band aligns with the base band.

In the case of Fig. 3b and 3e, the full data is shown in Fig. S5.2 below. In this case we also show two sets of three lines (for $\Delta E = 0$ eV, $\Delta E = 0.1$ eV, and $\Delta E = 0.2$ eV): the first set is for stronger intra-band scattering (dashed lines), whereas the second set is for stronger inter-band scattering (solid lines). In the case of a light aligned band (left column), what contributes to the changes in the PF are primarily changes in the conductivity, whereas the Seebeck coefficient shows less variations (some variations at high Fermi levels are observed as indicated in the zoomed-in inset). In the case where we consider stronger intra-band scattering (dashed lines), an increase in the conductivity upon band alignment is observed, which translates to PF improvements, whereas in this case the Seebeck coefficient slightly decreases upon band alignment (at high Fermi levels – see inset). In the case where we consider stronger inter-band scattering (solid lines), PF reduction is observed upon band alignment, and this reduction originates again from the conductivity reductions (compare the black to the fully aligned red line). Following the expected inverse trend, in this case the Seebeck coefficient increases slightly upon full alignment, but not enough to result to PF improvements (see higher red line in inset). In the second case (right column), when considering heavy band alignment, upon stronger intra-band scattering (dashed lines) the PF is degraded only slightly upon band alignment. In this case neither the conductivity nor the Seebeck coefficient experience noticeable changes. In the case of stronger inter-band scattering, however, the PF is strongly reduced upon band alignment (solid lines – red is lower than the black one). Again, in this case it is the conductivity which is reduced noticeably. The Seebeck coefficient increases slightly (inset - compare the black line to the fully aligned case shown by the red line), but still the conductivity is what dominated the PF reduction.

So overall, the changes in the PF are controlled by changes in the conductivity, rather that the Seebeck coefficient.

We have to stress, however, that upon performing computational studies, one has to choose what quantity will be represented in the x-axis, and that has consequences on the phenomenological shifts of the quantities on the x-axis, but not on the PF shape or the PF peak, other than a shift in the x-axis of the plots.

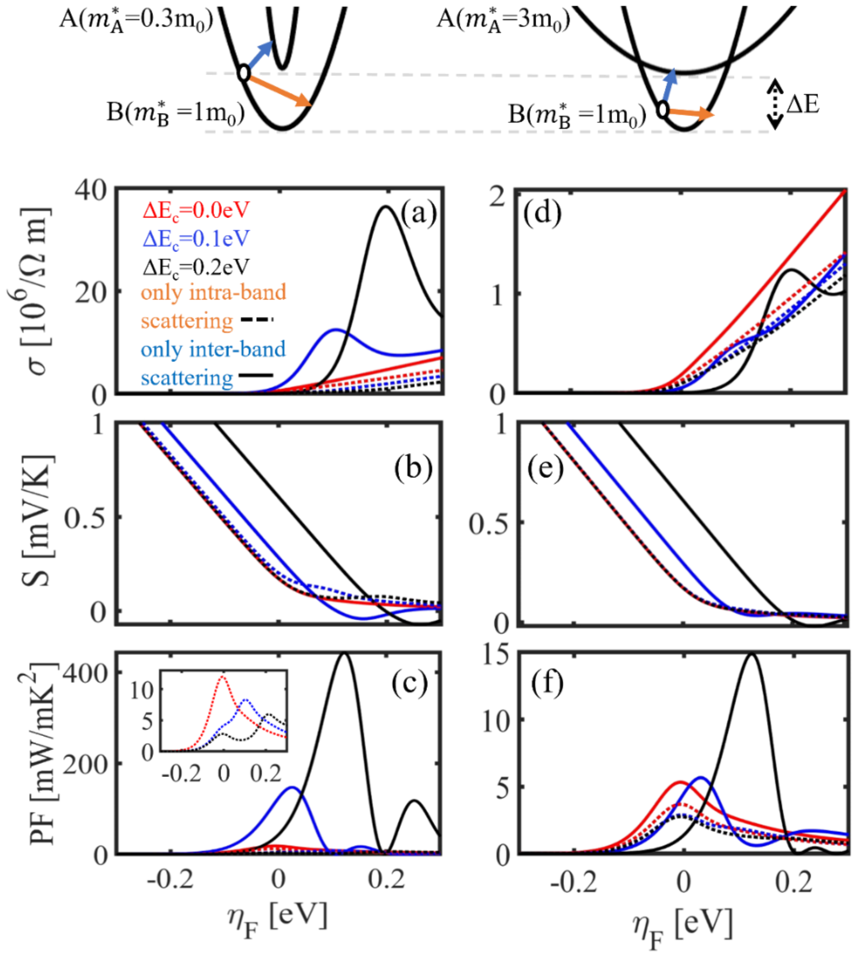

Figure S5.1: Full data lines for Fig. 2 of the main manuscript. Showing σ, S and PF for light and heavy band alignment.

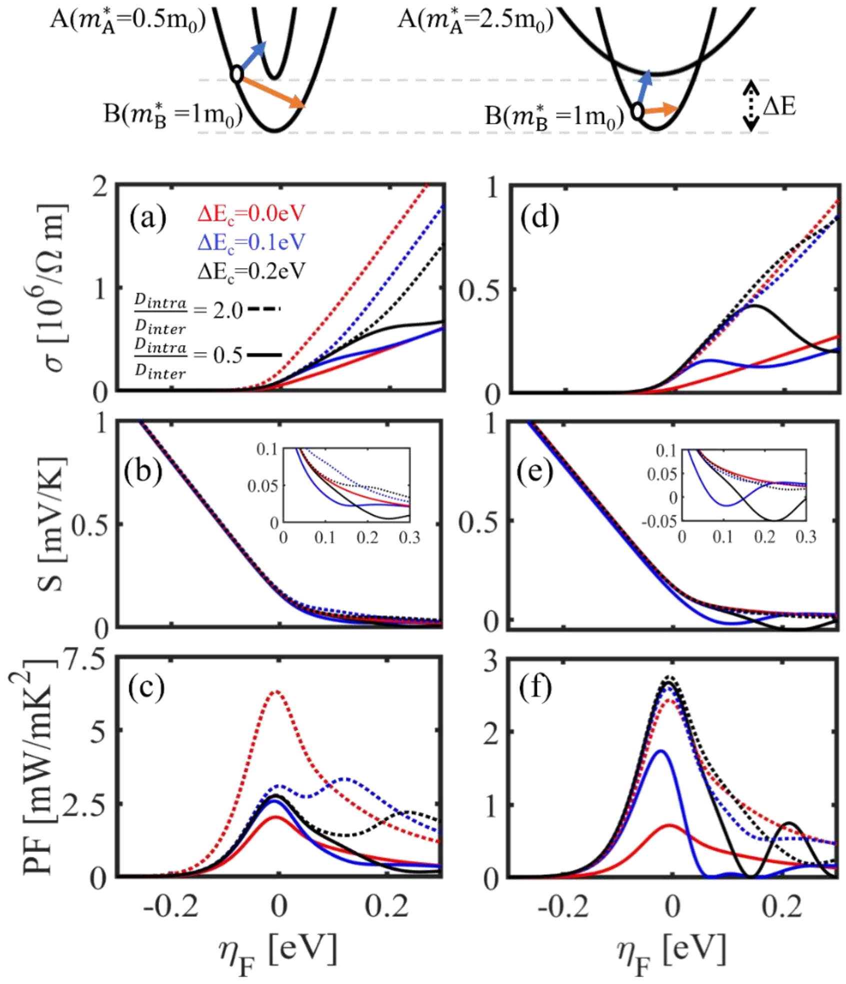

Figure S5.2: Full data lines for Fig. 3b (left column) and Fig. 3e (right column) of the main manuscript.

Section 6: Altering the base band effective mass

In our simulations we kept the mass of the base valley at 1 m_0 at all cases to keep the number of possible parameters that we vary at a minimum. What matters finally in our conclusions is the relative mass variation between the base valley effective mass and the aligned valley effective mass. Thus, if we lower the effective mass of the base band, we will require to align even lighter upper valley to achieve the same PF improvement ratio. With regards to our overall conclusion, that the composite valley system we align needs to be of higher conductivity compared to the base valley, it follows again that a lighter base band will require a more

conductive aligned valley system for PF improvements, whereas with a heavier base mass we can afford to align less conducting valleys.

Below we illustrate this with relevant simulations, in which we keep the aligned band mass constant as shown in Fig. S6 and vary the base band mass. In the first case (first row) we start with the case presented in Fig. 1a in the main paper, where a PF increase of 33% is observed for $m_B = 1\ m_0$. We then increase the mass of the base band. As the base band mass increases the overall PF amplitude decreases since now the heavy base band degrades conductivity. Upon aligning the upper light band in these heavier base band cases, a similar improvement is observed. This is expected. At band alignment the light band will tend to provide a certain boost in the PF on top of the heavy base band in absolute terms. But scattering into the heavy band increases as the base band mass increases and the conductivity of the aligned band reduces in absolute terms. On the other hand, the conductivity of the base band and its PF is also reduced in absolute terms. Thus, there is a lower PF starting point in the case of heavy base bands, and a lower PF increase from the light aligned band contribution, thus overall, the relative PF increase remains similar when we change the base band mass. This pretty much follows the pink line in Fig. 4 of the main paper (in the left half of the figure with m_A/m_B from 0.3 down to 0.15), where the PF improvements tend to saturate with the m_A/m_B ratio.

In the second case (second row), we consider a heavy mass aligned band, which we keep constant, and increase the mass of the base band. The most left case is the one presented in Fig. 1b of the main paper, where a 55% reduction in the PF is observed (aligning a heavy band). As the base band mass increases, the overall PF amplitude decreases since now the heavy base band degrades conductivity. The relative PF reduction from the mis-aligned to the fully aligned case however, is reduced in percentage. This is also expected. The black line, mis-aligned case is dominated by the lower base band for which the PF is reduced as the base band mass increases. Once a heavy band is aligned with the light base band in the first left-most case, the conductivity of the base band suffers significantly (large relative additional scattering rate introduced), and a larger PF reduction is observed. As the base band mass increases, its conductivity suffers relatively less (i.e. the additional scattering rate introduced is relatively less and less as the base band becomes heavier), and the PF is degraded less in relative terms. This is pretty much following the pink line in Fig. 4 of the main paper (in the right half of the figure with m_A/m_B from 3 down to 1.5).

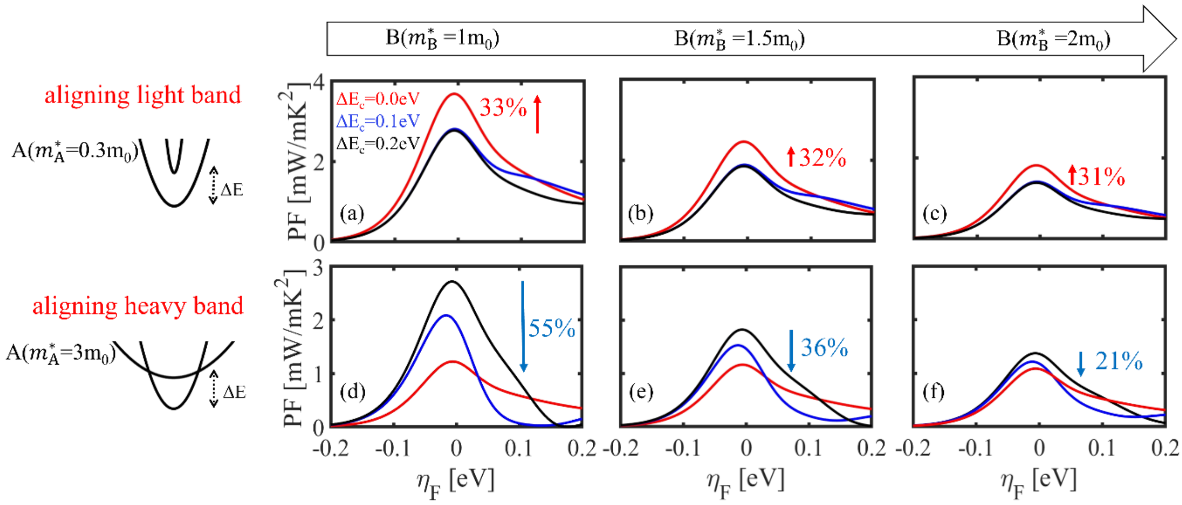

Figure S6.1: Simulations considering changes in the base band mass, rather than the aligned band mass.

References

[1] Jain, A.; Ong, S.P.; Hautier, G.; Chen, W.; Richards, W. D.; Dacek, S.; Cholia, S.; Gunter, D.; Skinner, D.; Ceder, G.; Persson, K. A. Commentary: The Materials Project: A materials genome approach to accelerating materials innovation. *APL Mater.* **2013**, 1(1).
